# Bacterial Ligands Generated in a Phagosome Are Targets of the Cytosolic Innate Immune System

**DOI:** 10.1371/journal.ppat.0030051

**Published:** 2007-03-30

**Authors:** Anat A Herskovits, Victoria Auerbuch, Daniel A Portnoy

**Affiliations:** 1 Department of Molecular and Cell Biology, University of California, Berkeley, California, United States of America; Massachusetts General Hospital and Harvard Medical School, United States of America; 2 School of Public Health, University of California, Berkeley, California, United States of America

## Abstract

Macrophages are permissive hosts to intracellular pathogens, but upon activation become microbiocidal effectors of innate and cell-mediated immunity. How the fate of internalized microorganisms is monitored by macrophages, and how that information is integrated to stimulate specific immune responses is not understood. Activation of macrophages with interferon (IFN)–γ leads to rapid killing and degradation of Listeria monocytogenes in a phagosome, thus preventing escape of bacteria to the cytosol. Here, we show that activated macrophages induce a specific gene expression program to L. monocytogenes degraded in the phago-lysosome. In addition to activation of Toll-like receptor (TLR) signaling pathways, degraded bacteria also activated a TLR-independent transcriptional response that was similar to the response induced by cytosolic L. monocytogenes. More specifically, degraded bacteria induced a TLR-independent IFN-β response that was previously shown to be specific to cytosolic bacteria and not to intact bacteria localized to the phagosome. This response required the generation of bacterial ligands in the phago-lysosome and was largely dependent on nucleotide-binding oligomerization domain 2 (NOD2), a cytosolic receptor known to respond to bacterial peptidoglycan fragments. The NOD2-dependent response to degraded bacteria required the phagosomal membrane potential and the activity of lysosomal proteases. The NOD2-dependent IFN-β production resulted from synergism with other cytosolic microbial sensors. This study supports the hypothesis that in activated macrophages, cytosolic innate immune receptors are activated by bacterial ligands generated in the phagosome and transported to the cytosol.

## Author Summary

Innate immune recognition of microorganisms has a direct impact on the type and the magnitude of the immune response elicited. While recognition of microorganisms relies on receptors that sense pathogen-associated molecular patterns, (PAMPs), it was reasonable to suspect that immune cells could discriminate between live and dead bacteria. Listeria monocytogenes is an intracellular pathogenic bacterium used extensively as a model system for studying basic aspects of innate and acquired immunity. L. monocytogenes is internalized by macrophages, escapes from a vacuole, multiplies within the cytosol, and spreads from cell to cell without lysing the cells. We used wild-type and bacterial mutants of L. monocytogenes to demonstrate that macrophages not only respond differently to bacteria that are growing in the cytosol and to non-growing bacteria that are trapped in a vacuole, but that they also can discriminate between intact or degraded bacteria in the vacuole. We showed that macrophages induce specific immune response when bacteria are killed and degraded. This response was directly correlated to the ability of macrophages to degrade bacteria and involved receptors that were located in the host cell cytosol. These observations led us to suggest that bacterial degradation products may serve as messengers that inform immune cells that bacteria were killed and degraded. This information might affect directly the immune response, for example, by down-regulating inflammatory responses that can be deleterious. We call these bacterial degradation products PAMP-PM (PAMP–post-mortem).

## Introduction

Macrophages are highly phagocytic cells that can act as benign scavengers, sentinels of microbial infection, and hosts to intracellular pathogens [1]. However, a key property of macrophages is their capacity to be immunologically activated by cytokines such as interferon (IFN)–γ. Subsequent to phagocytosis of microorganisms, activation is manifested as an enhanced microbiocidal, degradative, and secretory capacity concomitant with maturation of phagosomes into acidic hydrolytic compartments [2]. How macrophages couple microbiocidal and degradative activity with the development of an appropriate immune response is critical to understanding the regulation of inflammation.

Recognition of microorganisms by the innate immune system is mediated by invariable pattern recognition receptors (PRRs) that bind conserved molecules present on microorganisms, referred to as pathogen-associated molecular patterns (PAMPs). Among PRRs are the Toll-like receptors (TLRs), type I integral membrane proteins located at the cytoplasmic membrane and internal membrane-bound compartments, and nucleotide-binding oligomerization domain (NOD) proteins located in the cell cytosol [3,4]. Microbial structures exposed on the bacterial cell surface, such as lipopolysacaccharide (LPS), peptidoglycan (PGN), and flagellin, are recognized by TLR4, 2, and 5, respectively, which are localized to the host cell surface. In contrast, microbial nucleic acids are recognized by TLR3, 7, and 9, which are located within intracellular membrane-bound compartments that can fuse with phagosomes during their maturation. Treatments of cells with agents that block vacuolar acidification abrogate responses mediated by TLR3, 7, and 9 [5,6]. Whereas TLRs detect microorganisms extracellularly or within the luminal side of the phagosome, the NOD-like receptor family may comprise a surveillance system that recognizes intracellular pathogens, leading to both transcriptional responses and activation of the inflammasome [7]. Among the cytosolic innate immune receptors, RIG-I and MDA5 recognize double-stranded RNA, whereas NOD1 and NOD2 recognize bacterial PGN degradation products [7–12].

Engagement of innate immune receptors with specific microbial ligands results in signaling pathways that culminate in host transcriptional responses associated with inflammation. Signaling pathways are characterized by their shared adaptor molecules. For example, MyD88 is a major adaptor that mediates immune responses downstream of all TLRs except TLR3, leading primarily to activation of nuclear factor (NF)–κB [13]. Interaction of MyD88 with TLR7 and 9 can also lead to the induction of type I IFN response through activation of interferon regulatory factor (IRF) 3/7. TLR3 and TLR4 can both induce type I IFN responses via another adaptor molecule, called Trif (Lps2), again through the activation of IRF3 [13,14]. The cytosolic receptors that recognize RNA and DNA signal by interacting with the mitochondrial membrane adaptor MAVS/VISA/IPS-1/Cardif [15], leading to activation of IRF3 and production of IFN-β. Less is known about the signaling pathways downstream of the NOD proteins, although NOD1 and NOD2 interact with the adaptor molecule Rip2 (RICK) to activate NF-κB [16].

Type I IFNs have been studied extensively with regard to their role as anti-viral cytokines, but their role in response to bacterial infection has been less studied, although bacterial LPS derived from Gram-negative bacteria is clearly an inducer of type I IFN [17]. Recently, it was shown that a Gram-positive pathogen, *Listeria monocytogenes,* induces type I IFN, but only upon entry into the host cell cytosol [18–21]. Mutants lacking the secreted pore-forming protein listeriolysin O (LLO) fail to escape from a phagosome and fail to induce IFN-β. Recognition of LLO-minus L. monocytogenes in the phagosome is largely MyD88-dependent, while recognition of cytosolic bacteria is MyD88-independent and IRF3-dependent [18–21]. The nature of the bacterial ligand(s) and the host PRRs responsible for the activation of IRF3 in response to cytosolic L. monocytogenes are not known, although bacterial DNA can recapitulate this response [22]. The production of type I IFN in response to L. monocytogenes is enigmatic, as mice lacking the IFNα/β receptor are more resistant to listeriosis [23–25].

A role of type I IFNs in the induction of acquired immunity has become increasingly recognized, and it has been suggested that a key feature of effective adjuvants is the capacity to induce type I IFN [26]. However, NOD2, the target of one of the most powerful adjuvants (muramyl dipeptide [MDP] derived from bacterial PGN), has not been associated with the expression of type I IFNs. In this report, we show that activated macrophages express IFN-β after phagocytosis and degradation of *L. monocytogenes,* and that NOD2 is necessary for full expression. IFN-β induction by LLO-minus bacteria was dependent on the activity of the macrophage vacuolar ATPase, not for acidification of the phagosome but for the generation of the phagosomal membrane potential which, we hypothesize, has a role in the active transport of bacterial ligands into the cytosol.

## Results

### Bacteria Degraded in the Phagosome Trigger Distinct Transcriptional Responses in IFN-γ–Activated Macrophages

**Figure 1:**
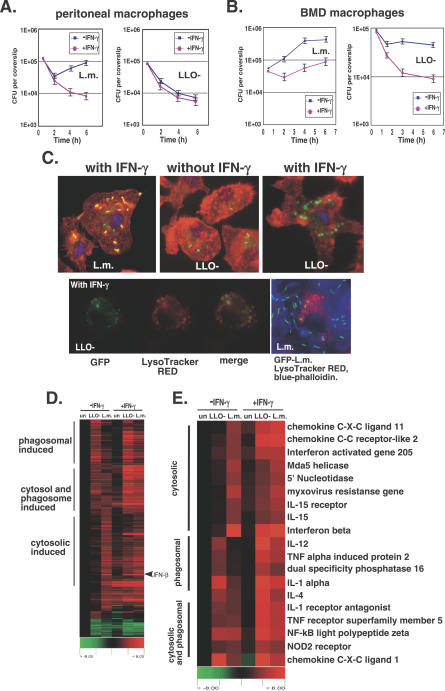
L. monocytogenes Is Killed and Degraded in IFN-γ–Activated Macrophages, Leading to a Specific Activation Program (A) Intracellular growth curve of w.t. L. monocytogenes (L.m.) and LLO-minus mutant (LLO-) in IFN-γ–activated (•) versus non-activated () resident peritoneal macrophages. (B) Intracellular growth curve of w.t. L. monocytogenes and LLO-minus mutant in IFN-γ–activated (•) versus non-activated () BMD macrophages. (C) Immunofluorescence microscopy of BMD macrophages infected with GFP-expressing, w.t. L. monocytogenes and LLO-minus mutant at 4 h.p.i. Macrophage actin was stained in red with rhodamine-phalloidin. Lower panel, staining of acidic vesicles with LysoTracker RED in LLO-minus and w.t. L. monocytogenes infections (w.t. infection is also stained with cuomarin-phalloidin for actin labeling in blue). Immunofluorescence data are representative of 90% of the infected macrophages in two independent experiments. (D) Macrophages transcriptional response to w.t. L. monocytogenes and LLO-minus mutant infection with and without activation of IFN-γ. Only the most changed genes upon bacterial infection of non-activated macrophages are presented (253 genes vary 5.6-fold and up). Genes were clustered using Pearson hierarchical clustering. All columns were normalized to uninfected macrophages (un) without IFN-γ treatment. Rows colorimetrically represent expression ratios of individual genes as describe in material and methods. (E) Presentation of selected genes (cytosolic-induced, phagosomal-induced, and both) and their expression profile in activated and non-activated macrophages infected with w.t. L. monocytogenes and LLO-minus bacteria.

It was previously demonstrated that macrophages respond differently to bacteria located in their cytosol (e.g., wild-type [w.t.] L. monocytogenes), compared to bacteria trapped in a phagosome (e.g., LLO-minus L. monocytogenes) [18,19]. Cytosolic bacteria trigger an MyD88-independent production of IFN-β, whereas phagosomal bacteria do not [20,21]. However, these observations were based on the response of macrophages that were not activated and consequently weakly bacteriocidal. In vivo, during L. monocytogenes infection, cytokines such as IFN-γ act on macrophages to render them highly bacteriocidal and therefore less permissive for L. monocytogenes replication [27]. We reasoned that the bacteriocidal activity of macrophages, such as killing and degradation of bacteria, would directly affect the innate immune response to L. monocytogenes infection. In order to test this hypothesis, we studied the response of IFN-γ–activated macrophages to infection with w.t. L. monocytogenes and an LLO-minus mutant. The bacteriocidal activity of macrophages was best demonstrated when peritoneal macrophages were infected with L. monocytogenes (Figure 1A). Growth curves of L. monocytogenes in activated peritoneal macrophages showed dramatic killing of w.t. bacteria. The number of bacteria recovered from the activated peritoneal macrophages decreased during 6 h of infection, while in non-activated peritoneal macrophages, bacteria were subjected to initial killing, but survivors continued to grow (Figure 1A). Infection of peritoneal macrophages with the LLO-minus mutant resulted in killing of bacteria even without IFN-γ treatment (Figure 1A). Unlike in peritoneal macrophages, the bacteriocidal activity of bone marrow–derived (BMD) macrophages was less profound and was completely dependent on IFN-γ treatment. Growth curves of w.t. L. monocytogenes in IFN-γ–activated BMD macrophages showed that IFN-γ treatment initially restricted the growth of w.t. *L. monocytogenes,* although bacteria were still able to escape to the cytosol and replicate (Figure 1B). The bactericidal activity of BMD macrophages was best observed when the cells were infected with the LLO-minus mutant. Like in peritoneal macrophages, a one-log decrease in the number of LLO-minus bacteria was recovered after 6 h of infection (Figure 1B). Since BMD macrophages killed phagosomal-trapped bacteria only upon IFN-γ activation, and w.t. L. monocytogenes were still able to escape to the cytosol in activated BMD macrophages, we chose to use these cells to study further the effect of the bacteriocidal activity on the innate immune response to L. monocytogenes. To examine whether L. monocytogenes were subjected to lysis in phagosomes, activated BMD macrophages were infected with w.t. L. monocytogenes or an LLO-minus mutant expressing cytosolic–green fluorescent protein (GFP). Immunofluorescence microscopy revealed that at 6 hours post-infection (h.p.i.), most w.t. bacteria were cytosolic, as many of them were engaged with actin tails, showed by co-localization of the GFP bacteria with the actin marker rhodamine-phalloidin. Only a few w.t. bacteria were labeled just with GFP, suggesting that they were trapped in the phagosome (Figure 1C). Infection with a GFP-expressing LLO-minus mutant revealed that non-activated macrophages contained one to two intact GFP-expressing bacteria per cell, whereas in activated macrophages the GFP was released from the bacteria and distributed in multiple vacuoles around the cell (Figure 1C). While we don't know the precise composition of these vacuoles, co-localization of some of the GFP-labeled vacuoles with the pH-sensitive dye LysoTracker RED suggested that they might have originated from the primary phagosome during its maturation to a phago-lysosome (Figure 1C). As a control, w.t. L. monocytogenes did not localize with LysoTracker RED labeling (Figure 1C). These results indicated that macrophage activation led to an enhanced degradative activity and trafficking of host-generated bacterial ligands, which potentially can be sensed by the innate immune system.

**Figure 2:**
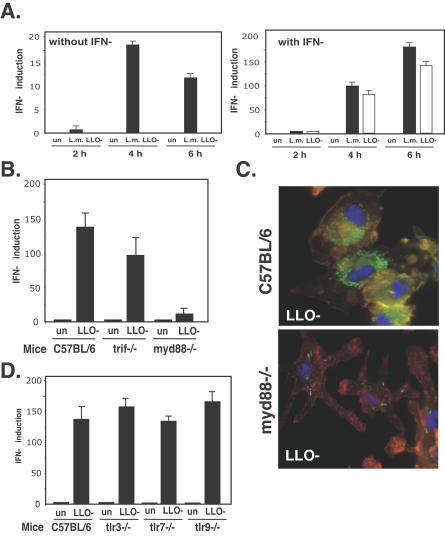
IFN-β Expression by LLO-Minus Mutant Is independent of TLR3, 7, and 9 (A) Time course analysis of *ifnβ* induction by w.t. L. monocytogenes (L.m.) and LLO-minus mutant (LLO-) in non-activated and IFN-γ–activated BMD macrophages. Analysis of IFN-β mRNA by Q-RT-PCR at 2, 4, and 6 h.p.i. un, uninfected macrophages. (B) Analysis of *ifnβ* induction in response to the LLO-minus mutant after infection of MyD88- and Trif-deficient activated BMD macrophages by Q-RT-PCR at 6 h.p.i. (C) Immunofluorescence microscopy of MyD88 and C57BL/6 activated BMD macrophages infected with the GFP-expressing LLO-minus mutant at 6 h.p.i. (D) Analysis of *ifnβ* induction in response to the LLO-minus mutant after infection of TLR3-, 7-, and 9–deficient activated BMD macrophages by Q-RT-PCR. Data correspond to the mean ± standard error of the mean (s.e.m.) (triplicate determinations) and are representative of three or more independent experiments. Immunofluorescence data are representative of 90% of the infected macrophages in two independent experiments.

In order to study the innate immune response of activated macrophages to L. monocytogenes infection, we used Mouse Exonic Evidence Based Oligonucleotide (MEEBO) microarrays [28]. Analysis of the gene expression profiles of IFN-γ–activated and non-activated BMD macrophages infected with w.t. L. monocytogenes and an LLO-minus mutant were performed. Consistent with previous studies, the response of non-activated macrophages to infection with w.t. L. monocytogenes and the LLO-minus mutant clustered into three groups of genes: i) genes that are largely induced by w.t. cytosolic bacteria (i.e. cytosolic-induced genes), ii) genes that are largely induced by phagosomal LLO-minus bacteria (i.e., phagosomal-induced genes), and iii) genes that are induced by both cytosolic and phagosomal bacteria (Figure 1D; 253 genes vary > 5.6-fold). Analysis of these genes in IFN-γ–activated macrophages infected with w.t. or LLO-minus bacteria reveled that many of the genes that were cytosolic or phagosomal-specific in non-activated macrophages were induced by both bacteria in IFN-γ–activated macrophages (Figure 1D). As expected, w.t. L. monocytogenes triggered expression of genes from the “phagosomal-specific genes” category in the activated macrophages. Under these conditions, some w.t. bacteria failed to escape to the cytosol and remained trapped in the phagosome; consequently, they induced phagosomal-specific genes as well (Figure 1B and 1C). Pro-inflammatory cytokines such as interleukin (IL)–12 and IL-1α, which are normally induced by LLO-minus bacteria, were highly induced by w.t. cytosolic bacteria in activated macrophages compared to non-activated macrophages (Figure 1E). Conversely, LLO-minus bacteria induced many “cytosolic-specific genes” upon activation of macrophages, including the most highly induced gene in the macrophages' response to w.t. *L. monocytogenes,* IFN-β (Figure 1E). Like IFN-β, other cytosolic-specific genes that are normally induced by w.t. bacteria, such as IL-15, chemokine CXC ligand 11, chemokine C-C receptor like 2, and type I IFN–related genes, were also induced by LLO-minus bacteria in activated macrophages (Figure 1E). Interestingly, IFN-β was among the 20 most induced genes in activated macrophages infected with LLO-minus mutant (out of 12,344 genes total), together with Nos2, ubiquitin D, and C-C receptor like 2 (Table S1). Since the IFN-β response to w.t. L. monocytogenes is well established, we were interested in whether the IFN-β response to LLO-minus bacteria shares common signaling pathways. Further validation of IFN-β induction by the LLO-minus mutant in activated macrophages was performed using quantative real-time PCR (Q-RT-PCR) analysis. A time course analysis of *ifnβ* expression during w.t. L. monocytogenes infection demonstrated that *ifnβ* induction increased 10-fold in activated macrophages compared to non-activated macrophages (Figure 2A). Upon infection with an LLO-minus mutant, activated macrophages induced *ifnβ* to the same level as in response to w.t. L. monocytogenes (Figure 2A). This was also the case when activated peritoneal macrophages were infected with the LLO-minus mutant (Figure S1). These results demonstrated that bacteria trapped in the degradative phago-lysosomes of activated macrophages trigger the induction of IFN-β, a response seen in non-activated macrophages only by bacteria able to access the cytosol. An obvious consequence of bacterial degradation is the release of bacterial ligands, such as nucleic acids, that are not normally exposed by either live bacteria or killed, but non-degraded, bacteria. It is possible that these bacterial degradation products triggered the induction of IFN-β by activated macrophages infected with LLO-minus mutant.

Since several TLRs are known to induce IFN-β through the adaptor molecules MyD88 and Trif, we used macrophages isolated from mice lacking each one of these adaptors to examine their role in the IFN-β response to degraded bacteria. Whereas *ifnβ* induction was slightly reduced in *Trif*-deficient macrophages, it was completely abolished in MyD88-deficient macrophages (Figure 2B). However, examination of IFN-γ–activated *myd88^−/−^* macrophages infected with LLO-minus GFP-expressing mutants revealed a defect in bacterial degradation. While in C57BL/6 macrophages, bacteria were lysed as shown by GFP distribution, in MyD88-deficient macrophages the LLO-minus mutant remained intact even 6 h.p.i (Figure 2C). Growth curves of the LLO-minus mutant in MyD88-deficient activated macrophages revealed a slight decrease in bacterial colony-forming units (CFUs) when compared to non-activated macrophages, suggesting that MyD88-deficient macrophages have a defect in killing of the LLO-minus mutant (Figure S1). This result, although striking, made it impossible to decipher the precise role played by MyD88 in the *ifnβ* induction by LLO-minus mutants (see discussion below).

Since IFN-β induction occurs downstream of TLR3, 7, and 9, which are involved in nucleic acid recognition, we examined the possibility that bacterial nucleic acids, possibly released upon bacterial degradation, trigger IFN-β production in activated macrophages. Macrophages isolated from mice lacking individual TLR3, 7, and 9 were infected with the LLO-minus mutant to test their involvement in bacterial nucleic acid recognition. Somewhat surprisingly, none of these TLRs had any detectable affect on the induction of *ifnβ* by LLO-minus L. monocytogenes (Figure 2D).

### NOD2 Detects Bacterial Ligands Generated in the Phagosomes

**Figure 3:**
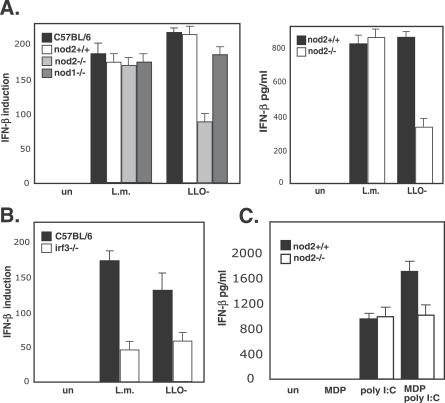
Role of NOD2 in the IFN-β Response to L. monocytogenes in Activated Macrophages (A) Analysis of *ifnβ* induction by Q-RT-PCR and IFN-β secretion (ELISA) by L. monocytogenes (L.m.) and the LLO-minus mutant (LLO-) in activated, NOD2-, and NOD1-deficient BMD macrophages. The level of induction in NOD2^−/−^ was compared to macrophages from w.t. NOD2 mice littermates. un, uninfected macrophages. (B) Analysis of *ifnβ* induction (Q-RT-PCR) by L. monocytogenes and the LLO-minus mutant in activated, IRF-3–deficient BMD macrophages in comparison to C57BL/6 macrophages. un, uninfected macrophages. (C) ELISA analysis of IFN-β amounts secreted by NOD2-deficient BMD macrophages in response to 100 μg/ml MDP and 100 μg/ml poly(I:C) delivered with lipofectamine. Data correspond to the mean ± s.e.m (triplicate determinations) and are representative of three or more independent experiments.

Since none of the TLRs seemed to be playing a role in the detection of LLO-minus *L. monocytogenes,* we considered other host receptors that recognize bacterial ligands. NOD1 and NOD2 are cytosolic proteins that are activated by muropeptides derived from bacterial PGN [29]. Surprisingly, NOD2 was involved in production of IFN-β in response to the LLO-minus mutant, as macrophages from NOD2-minus mice expressed and secreted 50% of IFN-β (Figure 3A). The induction of IFN-β by w.t L. monocytogenes or an LLO-minus mutant was independent of NOD1 (Figure 3A). Although *nod2* gene expression was induced by both w.t L. monocytogenes and LLO-minus bacteria (Figure 1D), it affected only the IFN-β response to phagosomal bacteria (LLO-minus) and not to cytosolic w.t. bacteria (Figure 3A)*.* Unlike NOD2, the transcriptional regulator IRF3 was required for IFN-β production by both vacuolar and cytosolic bacteria, suggesting that both pathways share common adaptors downstream of the signaling pathways leading to type I IFN response (Figure 3B).

This is the first report to our knowledge that links NOD2 activation with type I IFN responses. MDP, the well-studied ligand of NOD2, does not induce IFN-β when delivered to the cytosol (Figure 3C). However, as NOD2 synergizes with other receptors for cytokine production [30–34], we tested the possibility that the IFN-β production in response to degraded bacteria was a result of synergism between NOD2 and other innate immune receptors. MDP was delivered with poly(I:C) (a dsRNA analog that is sensed by phagosomal and cytosolic receptors) to the activated macrophage cytosol. Whereas poly(I:C) alone led to production of IFN-β, when combined with MDP, the amount of IFN-β secreted by macrophages was 40% higher than with poly(I:C) alone (Figure 3C). This increase in IFN-β production was dependent on NOD2, demonstrating that NOD2 can synergize with other receptors leading to an enhanced type I IFN response.

### Degradation of Bacteria and Phagosomal Membrane Potential Are Required for the Induction of IFN-β

**Figure 4:**
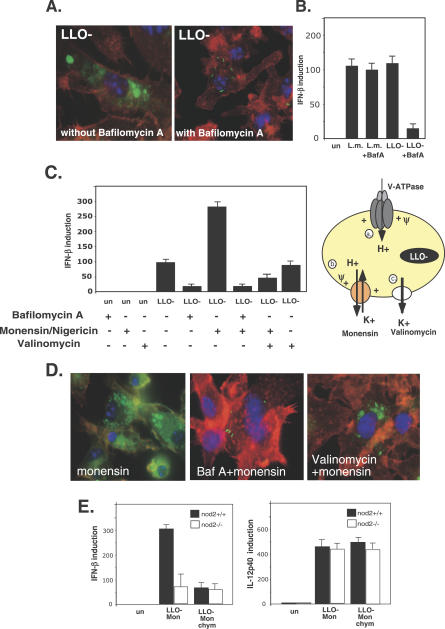
Effects of Phagosome Acidification and Phagosomal Membrane Potential on IFN-β Expression (A) Immunofluorescence microscopy of C57BL/6 BMD macrophages infected with GFP-expressing LLO-minus mutant (LLO-) with and without bafilomycin A treatment (6 h.p.i.). (B) Q-RT-PCR analysis of *ifnβ* induction in response to L. monocytogenes (L.m.) and the LLO-minus mutant with and without bafilomycin A treatment (BafA). Bafilomycin A was added 30 min post-infection. (C) Effect of the ionophors monensin, nigericin, and valinomycin on IFN-β response to the LLO-minus mutant, analyzed by Q-RT-PCR. Schematic presentation of the phagosome, ionophor mode of action, and H^+^ and K^+^ ion transfer across the phagosomal membrane. (a.) V-ATPase, which generates an electrochemical potential (ψ), inside positive and acidic. (b.) Monensin or nigericin, which establish a chemical K^+^ gradient by electroneutral H^+^/K^+^ exchange (c.) Valinomycin, which dissipates the electrical potential by electrogenic efflux of K^+^. (D) Immunofluorescence microscopy of C57BL/6 BMD macrophages infected with GFP-expressing LLO-minus mutant under ionophor treatments. All ionophors were added 30 min post-infection. (E) Effect of monensin (Mon) and chymostatin (Chym) on the IFN-β and IL-12p40 response to LLO-minus mutant in NOD2-deficient cells and NOD2 w.t. cells, analyzed by Q-RT-PCR. Data correspond to the mean ± s.e.m. (triplicate determinations) and are representative of three or more independent experiments. Immunofluorescence data are representative for 99% of the infected macrophages in two independent experiments.

Since the IFN-β response to phagosomal-degraded bacteria required the cytosolic receptor NOD2, we hypothesized that bacterial ligands were generated and transported from the phagosome and detected in the cytosol. In order to test whether degradation of bacteria is a prerequisite for IFN-β induction by LLO-minus mutants, we treated activated macrophages with bafilomycin A, a specific inhibitor of the vacuolar ATPase proton pump (V-ATPase). Bafilomycin A inhibits phagosome acidification and blocks maturation of phagosomes to phago-lysosomes. Bafilomycin A–treated macrophages indeed failed to degrade internalized LLO-minus mutants (Figure 4A), and did not induce *ifnβ* (Figure 4B), whereas this treatment had no effect on induction of *ifnβ* by cytosolic L. monocytogenes (Figure 4B). To distinguish between the requirement of degradation of bacteria or of acidification of the phagosome for IFN-β signaling, we used alternative endosomal acidification inhibitors, monensin or nigericin, which act differently than bafilomycin A. Monensin and nigericin are electro-neutral monovalent cation exchangers that are widely used to exchange K^+^/H^+^ ions across biological membranes [35]. In the presence of active V-ATPase, treatment with monensin or nigericin will induce intra-phagosomal accumulation of K^+^ ions as a result of exchange with luminal H^+^ (Figure 4C). This will lead to neutralization of vacuolar pH without changing the vacuolar membrane potential [35]. Neither addition of nigericin nor monensin blocked the induction of *ifnβ;* in fact, both resulted in the enhanced induction of *ifnβ* (Figure 4C), while neutralizing the phagosomal pH as determined by Lyso-Tracker RED staining (unpublished data). Combining bafilomycin A with monensin (or nigericin) or bafilomycin A alone abrogated *ifnβ* induction, demonstrating that the enhanced induction originated from acidic (phago-lysosome) compartments (Figure 4C). Immunofluorescence microscopy revealed that monensin treatment did not block phagosome maturation and bacterial degradation in activated macrophages except when combined with bafilomycin A (Figure 4D). These results demonstrated that while acidification of the phagosome was not required for IFN-β signaling, phagosome maturation and bacterial degradation were necessary for this response to LLO-minus mutants.

Since the V-ATPase contributes to the electrochemical potential across the phagosomal membrane [36], bafilomycin A treatment also results in dissipation of phagosomal membrane potential, which is not the case with monensin and nigericin [37]. We asked whether the phagosomal membrane potential was important for IFN-β signaling. In order to address this question, we dissipated the phagosomal membrane potential of monensin-treated cells by using the K^+^-specific ionophor valinomycin. Valinomycin transports K^+^ ions in accordance with existing chemical gradients, which together with monensin treatment results in leakage of accumulated K^+^ ions from the phagosome to the cytosol and, consequently, depolarization of the phagosomal membrane (Figure 4C). Treatment of valinomycin resulted in lower levels of *ifnβ* induction, although it did not affect degradation of bacteria (Figure 4C and 4D). These results suggest that the phagosomal membrane potential is required for IFN-β signaling in response to the LLO-mutant.

Next, we tested the role of NOD2 in the enhanced production of IFN-β by monensin treatment; interestingly, the induction of IFN-β was reduced by 4-fold in NOD2-deficient macrophages (Figure 4E). Possibly, monensin treatment impaired the signaling from TLRs that require acidic pH [5], leading to a greater effect on IFN-β expression in the NOD2-deficient macrophages. Importantly, NOD2 dependency was specific for a subset of cytokines like IFN-β, as other cytokines like IL-12p40, which are induced by TLRs, were unaffected by the NOD2 mutation (Figure 4E).

Recently, it was demonstrated that potassium ion flux plays a role in the activation of several proteases in the phagocytic vacuole of neutrophils [38]. Since treatment with monensin generates an influx of potassium ions into the phagosome, we were interested in whether lysosomal proteases play a role in the enhancement of IFN-β signaling by monensin treatment. In order to test this hypothesis, we treated cells with the protease inhibitor, chymostatin, which inhibits lysosomal serine and cysteine proteinases and several cathepsins. Chymostatin treatment resulted in a 4-fold decrease in the *ifnβ* expression and had no additional effect in NOD2-deficient macrophages (Figure 4E). Moreover, chymostatin treatment was also specific to *ifnβ* induction and had no effect on *il-12p40* induction (Figure 4E). These results suggest that bacterial lysis and further digestion by lysosomal proteases are required for generation of a NOD2 ligand, leading to *ifnβ* induction.

### L. monocytogenes PGN Is Degraded in the Phago-Lysosome and Can Induce NOD2-Dependent Signaling

**Figure 5:**
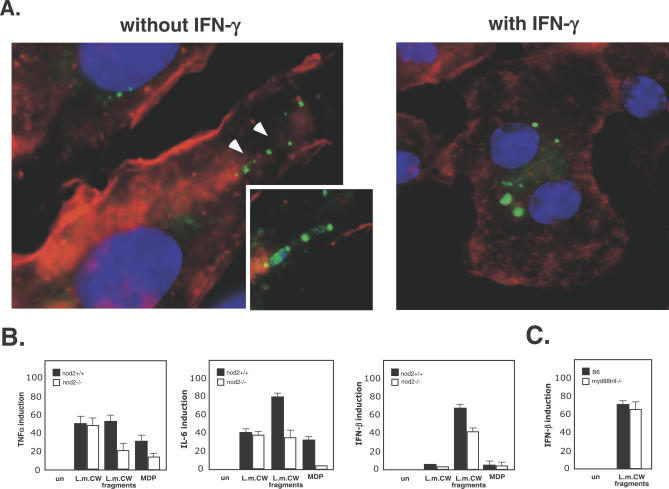
L. monocytogenes PGN Is Degraded in the Phago-Lysosome and Induces NOD2-Dependent Responses (A) Immunofluorescence microscopy of activated and non-activated BMD macrophages infected with FL-Van–labeled LLO-minus mutant. FL-Van labeling localized to bacterial poles (arrowheads and magnified picture). (B) Response of activated macrophages to L. monocytogenes CW preparation and CW degradation fragments (L.m.CW fragments) delivered with lipofectamine. Level of TNFα, IL-6, and IFN-β induction determined by Q-RT-PCR. (C) Induction of IFN-β response by L. monocytogenes CW fragments in Myd88/Trif double-knockout activated macrophages. Data correspond to the mean ± s.e.m. (triplicate determinations).

NOD2 is activated by MDP, but how MDP is generated and transported to the cytosol is unknown. The phago-lysosome contains enzymes that can potentially degrade PGN of bacteria, such as lysozyme [39]. However, while L. monocytogenes PGN is resistant to lysozyme cleavage [40], we asked whether it is still cleaved in the phago-lysosomes of activated macrophages. In order to address this question, we labeled the PGN of LLO-minus bacteria prior to infection with fluorescent vancomycin (FL-Van) that binds specifically to the terminal D-alanyl-D-alanine moieties of PGN [41]. Vancomycin labeling is localized to sites of nascent PGN synthesis, which results in polar bacterial staining (Figure 5A). Whereas in non-activated macrophages we could detect intact bacteria, in activated macrophages the FL-Van PGN labeling was distributed in large vacuoles, most likely due to bacterial cell wall (CW) breakdown (Figure 5A). The FL-Van PGN labeling was localized to acidic vacuoles determined by LysoTracker RED staining (not shown). While labeling of intracellular bacteria with FL-Van was not as efficient as the GFP labeling, we were able to detect large vacuoles (larger then a bacterial cell) in which the FL-Van labeling was equally distributed, suggesting that the bacterial PGN was released in these vacuoles.

Next, we examined whether L. monocytogenes PGN contains a NOD2 ligand and whether it can induce cytokines production in a NOD2-dependent manner. The CW fraction of L. monocytogenes was purified and treated with RNAse A and DNAse I to prevent nucleic acids contamination. While L. monocytogenes intact CW induces tumor necrosis factor (TNF) α and IL-6, that response was independent of NOD2 (Figure 5B). When the CW was degraded in vitro with the muramidase mutanolysin that specifically cleaves the PGN glycan backbone (NAG-NAM), the degradation products (like MDP) triggered TNFα and IL-6 induction, which was largely dependent on NOD2 (Figure 5B). The delivery of CW fragments and MDP to the macrophages cytosol was mediated by lipofectamine, which resulted in a 5-fold increase in the response to these ligands. Interestingly, only CW-derived fragments and not intact CW or MDP itself induce *ifnβ*. However, this induction was only partially (30%) dependent on NOD2 (Figure 5B). Since IFN-β expression was induced by purified bacterial CW fragments, we addressed the role of MyD88 and Trif in *ifnβ* induction. We found that neither of these adaptors was required for *ifnβ* induction, raising the possibility that cytosolic microbial sensors are involved in this response (Figure 5C). These results demonstrated that L. monocytogenes PGN contains a NOD2 ligand that becomes accessible only after degradation with a muramidase, and that it can induce a pro-inflammatory response when delivered to the cytosol. However, the exact nature of the PGN fragments generated in the phago-lysosome and the in vivo ligand recognized by NOD2 are not yet known.

## Discussion

We have investigated the relationship between two fundamental processes of macrophages, degradation of bacteria and induction of innate immune response. In vivo, IFN-γ has a major role in controlling L. monocytogenes infection. By activating immune cells such as macrophages, IFN-γ enhances the bacteriocidal activity of macrophages and renders them less permissive to L. monocytogenes replication. In this study, we used the LLO-minus mutant to ask whether the bacteriocidal activity of macrophages has a role in the innate immune response to L. monocytogenes infection. Here, we show that phagosomal-degraded bacteria induce a specific innate immune response that is different than the response to phagosomal intact bacteria. We found that phagosomal-degraded bacteria induced type I IFN, a response that was previously shown to be specific to intracellularly growing L. monocytogenes. This research report shows that the cytosolic receptor NOD2 enhances the induction of IFN-β by phagosomal-trapped *L. monocytogenes,* but only when these bacteria are killed and degraded in the phago-lysosomes of IFN-γ–activated macrophages. To our knowledge, this is the first study to demonstrate that bacterial breakdown products generated in the phago-lysosome are targets for intracellular innate immune sensors. This study suggests that induction of IFN-β in response to L. monocytogenes in vivo might result from two distinct signaling pathways, one of them largely dependent on NOD2 [20]. These results are consistent with a model in which bacterial breakdown products generated in the phagosome are transported to the cytosol, where they are detected by cytosolic microbial innate immune receptors.

NOD2 is activated by small muropeptides derived from bacterial PGN [29,42]. Although MDP (MurNAc-l-Ala-d-Gln) was shown to be the minimal motif recognized by NOD2, the natural ligand(s) sensed by NOD2 in vivo are not known. Mammals and bacteria contain muramidases, like lysozyme, or in the case of bacteria, lytic transglycosylases, that can generate muropeptide analogs of MDP such as GlcNAc-MurNAc-l-Ala-d-Gln [43]. However, only bacteria are known to possess the endopeptidases with the specificity to generate the NOD2 ligands. For example, L. monocytogenes secrets a highly expressed endopeptidase, p60, that cleaves the bond between D-Glu and *meso*-DAP, thus potentially generating NOD2 ligands [44]. In the phago-lysosome, L. monocytogenes is potentially subjected to degradation by both bacterial and host enzymes. We found that protease inhibitors blocked the NOD2-dependent response without affecting the TLR-dependent response. Therefore, lysosomal proteases might contribute to degradation of the bacterial CW, thereby facilitating further digestion of the PGN. A role for proteases was most evident upon H^+^/K^+^ ionophors treatment. Interestingly, a role for potassium influx and protease activation in neutrophils has been proposed by Reeve et al. [38]. These investigators have suggested that potassium influx into phagocytic vacuoles results in the release of cationic proteases from the anionic sulphated proteoglycan matrix, thus resulting in microbial degradation. During treatment with H^+^/K^+^ ionophors that cause a phagosomal potassium influx, we found that protease inhibitors blocked the IFN-β expression, but had no affect on expression of IL-12p40, suggesting that proteolysis is necessary to generate the ligands transported to the cytosol, but not those recognized in the phagosome. Taken together, these results show that phagosomal degradation of L. monocytogenes resulted in the NOD2-dependent production of IFN-β, presumably due to the production of the appropriate muramyl dipeptide(s) generated during bacterial degradation.

NOD1 and NOD2 are cytosolic proteins that are activated by small PGN fragments. Although the mechanism(s) that lead to PGN entry into the cytosol are not known, it was suggested that a specific transport system might be involved [45]. In the case of cytosolic pathogens such as Shigella flexneri or *L. monocytogenes,* it is possible that NOD1 and NOD2 recognize PGN fragments released during bacterial growth [46,47]. In addition, NOD1 and NOD2 may be activated by PGN fragments, introduced into host cells during infection by pathogens that possess auxiliary secretion systems. Indeed, activation of NOD1 occurred upon infection of epithelial cells by Helicobacter pylori [48]. A third hypothesis, consistent with our data, is that bacterial products leak or are actively transported from the phagosome to the cytosol [1]. An example for active transport of NOD2 ligands was demonstrated in epithelial cells where the peptide transporter hPepT1 was shown to transport MDP into the colonic epithelial cells, leading to NF-κB activation [45]. Although the mechanism of transport in macrophages is not known, here we present pharmacological evidence that the phagosomal membrane potential is crucial for NOD2-dependent IFN-β responses. While monensin and valinomycin treatments did not affect degradation of bacteria, addition of valinomycin to monensin-treated cells resulted in dissipation of the phagosomal membrane potential and reduction in IFN-β response. This observation leads to a hypothesis that the phagosomal membrane potential could have a direct role as a driving force for transport of ligands into the cytosol, or might be involved indirectly by affecting the function of membrane proteins involved in the transport process.

There are a number of well-characterized host signal–transduction pathways stimulated by viral and/or bacterial products that result in the production of type I IFN [17]. Intriguingly, DNA and RNA are recognized by both intracellular TLRs and by caspase recruitment domain (CARD)–containing cytosolic proteins, both leading to type I IFN production. Therefore, we initially hypothesized that the induction of IFN-β by degraded L. monocytogenes was likely associated with one of these TLRs, as bacterial nucleic acids are undoubtedly released into the phagosome upon bacteriolysis. However, none of the individual TLR knockouts had a measurable affect on the production of IFN-β, although it is still possible that there is an overlapping affect of individual TLRs. Recently, two cytosolic dsRNA helicases with CARD-domains, RIG-I and MDA5, have been identified and shown to lead to IFN-β production in response to viral nucleic acids by interacting with the mitochondrial membrane adaptor called MAVS/VISA/IPS-1/Cardif [15]. Interestingly, microarray data revealed that whereas MDA5 is induced only by w.t. L. monocytogenes in non-activated macrophages, it is induced by both w.t. and LLO-minus bacteria in activated macrophages (Figure 1D). Since L. monocytogenes DNA and RNA preparations were able to induce IFN-β when delivered to the cytosol (A. Herskovits, unpublished data; [22]), it is possible that cytosolic nucleic-acids sensors such as MDA5 and RIG-I are contributing to the expression of IFN-β in response to degraded L. monocytogenes.

This report has demonstrated that NOD2 activation enhances IFN-β production, and although we do not know the exact nature of this signaling pathway, since NOD2 contains two CARD domains, we speculate that it may be interacting with other CARD-containing adapters. Whereas purified MDP, the synthetic activator of NOD2, did not lead to IFN-β production, when combined with poly (I:C), it enhanced the induction of IFN-β in a NOD2-dependent manner. Our in vitro preparation of CW fragments recapitulated the induction of IFN-β when delivered to the macrophage's cytosol. However, this induction was only partially dependent on NOD2. Since NOD2 is known to act synergistically with other innate immune receptors [30–34], we suggest that the NOD2-dependent IFN-β response is a result of synergism between PGN fragments generated in phagosomes and other bacterial ligands exposed upon bacterial lysis. Interestingly, it was recently shown that delivery of L. monocytogenes DNA to the cytosol promotes IFN-β production [22], raising the possibility that release of bacterial nucleic acids in the cytosol might be involved in type I IFN response to degraded bacteria.

This study highlights the downstream consequences that result from the enhanced microbiocidal and degradative capacity of IFN-γ–activated macrophages. Whereas non-activated macrophages show some bacteriocidal capacity, only activated macrophages killed and degraded bacteria. Bacterial degradation has a number of potential immunological consequences. A well-established consequence of phagosomal degradation is the generation of bacterial peptides ligands, which leads to the development of major histocompatibility complex class II–dependent responses [49]. In this study, we show that digestion of bacteria in a phagosome results in induction of specific innate immune responses that differ from a response to intact bacteria. We demonstrated a direct correlation between bacterial degradation and macrophage expression of IFN-β. Macrophages that failed to degrade bacteria failed to express IFN-β. Blocking degradation of bacteria by bafilomycin A treatment resulted in loss of signaling. While the role of the V-ATPase in phago-lysosome maturation is well characterized, we found that the signaling adaptor MyD88 was also essential for bacterial degradation. MyD88-deficient macrophages failed to degrade bacteria or express IFN-β when presented with intact bacteria, but did express IFN-β when bacterial CW fragments were delivered directly to the cytosol. Although the role of MyD88 in phagosome maturation is controversial, it is possible that MyD88 is involved in the initial signaling pathways that promote macrophage activation and their capacity to kill and degrade bacteria. Indeed, it was shown that the induction of many genes by IFN-γ is dependent on MyD88 [50]. Recently, it was suggested that MyD88 is required for proper assembly of the phagosomal NADPH oxidase, thus affecting the killing of Gram-negative bacteria [51]. It is clear that immune signaling pathways affect cellular processes in specialized phagocytic cells, but how these processes, such as pathogen digestion and generation of new ligands, are involved in further shaping the immune response is less understood. We speculate that macrophages can discriminate between ligands that are presented by live bacteria or ligands that are generated after degradation of bacteria (post-mortem). We refer to these ligands as pathogen-associated molecular patterns post-mortem (PAMP-PM). The results of this study suggest that a fully active phagosome provides PAMP-PM for detection by the innate immune system. However, this “information” is not restricted to receptors that are located in the phagosome, but crosses the phagosomal membrane to activate intracellular immune receptors as well. Lastly, we suggest that bacterial degradation in the phagosome plays a major role in the development of innate and acquired immune responses.

## Materials and Methods

### Bacterial strains.

The L. monocytogenes strains used were a w.t. strain, 10403S and 10403S expressing GFP, or strains containing in-frame deletions of the *hly* gene (LLO, DP-L2161) [52] and DP-L2161 expressing GFP [53]. Single colonies were inoculated into 2 ml of BHI broth (brain-heart infusion) and incubated overnight at 30 °C without shaking.

### Mice.

C57BL/6 mice were obtained from The Jackson Laboratory (http://www.jax.org). CD-1 mice were obtained from Charles River Laboratory (http://www.criver.com). All knockout mice used in this study were on the C57BL/6 background or backcrossed with C57BL/6 mice for at least eight generations. Femurs or mice were obtained from the following source: *myd88^–/–^* from R. Medzhitov, Yale University School of Medicine, New Haven, Connecticut; *tlr3^−/−^*, *tlr7^−/−^*, *tlr9^−/−^* from K. A. Fitzgerald and D. Golenbock, University of Massachusetts Medical School, Worcester, Massachusetts; *trif^−/−^ (lps2/lps2), myd88trif^−/−^*, from B. Beutler, The Scripps Research Institute, La Jolla. California; *nod2^−/−^* [54] from V. Dixit, Genentech, South San Francisco, California; *nod1^−/−^* from Millennium, Cambridge, Massachusetts; *irf3^−/−^* from G. Cheng, Department of Microbiology, Immunology and Molecular Genetics, University of California, Los Angeles, California.

### Cell culture, infections, and treatments.

Primary cultures of resident peritoneal macrophages were prepared from CD-1 mice as previously described [27]. BMD macrophages were isolated from 6- to 8-wk-old female mice and cultured as described [55]. Activation of macrophages was done by incubating macrophage monolayer with 1 ng/ml recombinant murine IFNγ (Biosource, http://www.biosource.com) for 36 h before infection and during infection. Approximately 8 × 10^6^ w.t. L. monocytogenes or 1 × 10^8^ LLO-minus bacteria were used to infect 2 × 10^6^ macrophages cells seeded on a 60-mm petri dish. These numbers resulted in infection of one to two bacteria per cell in the case of w.t. *L. monocytogenes,* and ~25–50 bacteria per cell in the case of the LLO-minus mutant. Thirty minutes after addition of bacteria, macrophage monolayers were washed three times with PBS, and fresh medium was added. At 1 h.p.i., gentamicin was added to 50 μg/ml to limit the growth of extracellular bacteria. Unless indicated otherwise, infections were completed at 6 h.p.i, and further analyzed. Activation of macrophages was confirmed by visual inspection after infection with GFP-expressing bacteria (Figure 1C). Where indicated, 0.5 μM bafilomycin A (Sigma, http://www.sigmaaldrich.com), 1 μM monensin (Sigma), 0.1 μM nigericin (Sigma), 1 μM valinomycin (Sigma), were added at 30 min post-infection. The protease inhibitor chymostatin (100 μM) (Sigma), was added at the time of infection. Growth curves of L. monocytogenes in macrophages cells were performed as described earlier [27].

### Microarrays.

MEEBO microarrays were printed at the Center for Advanced Technology at University of California San Francisco [28]. Each microarray experiment was done in triplicate. Macrophages were infected with w.t. and LLO-minus mutant for 5 h with and without IFN-γ treatment. Then, macrophages were washed with PBS, and total RNA was extracted using RNAqueous kit (Ambion, http://www.ambion.com). A half microgram of RNA was amplified using Amino Allyl MessageAmp II aRNA Amplification Kit (Ambion). A total of 5 μg of amplified RNA from each sample was labeled fluorescently with Cy5 (Amersham, http://www.gelifesciences.com) and mixed with a Cy3 (Amersham)–labeled reference pool. The common reference pool contained equal amounts (5 μg of each) of amplified RNA from all the samples analyzed in the experiment (including uninfected, hly, and w.t. samples from activated and non-activated macrophages). RNA was hybridized to the MEEBO microarrays for 48 h. Microarrays were gridded using SpotReader software (Niles Scientific, http://www.nilesscientific.com) and GenePix Pro 6 (Molecular Devices, http://www.moleculardevices.com), and analyzed using Acuity 4 software (Molecular Devices). Highest quality spots meeting extra-stringent criteria were identified. These highest quality spots were used to calculate normalization factors such that the median Cy5/Cy3 ratio was brought to 1. These factors were then applied to the entire dataset after removing low quality spots. Finally, all arrays were normalized to the uninfected non-activated macrophages array. Significant analysis of microarrays (SAM) algorithm was used with two-class unpaired designs to identify genes that were differentially expressed in w.t. versus LLO-minus infection of non-activated macrophages, and in LLO-minus mutant infections of activated versus non-activated macrophages. Genes that showed a 5.6-fold or greater difference were selected for further analysis. Pearson hierarchical clustering was applied on selected genes.

### L. monocytogenes CW preparation and delivery.

One liter of L. monocytogenes culture was grown at 37 °C to exponential phase and used for CW preparation. Bacterial cells were lysed by three passages through a French press at 12,000 PSI, and treated with DNAse I (Invitrogen, http://www.invitrogen.com). After removal of cell debris, supernatant was added drop by drop to 8% boiling SDS with stirring, and was boiled for an additional 30 min [56]. The mixture was cooled overnight at room temperature, and washed with hot water. CW was washed in 0.1% Triton X-100, and then washed six times with water and stored at −20 °C. Mutanolysin treatment was done overnight in 50 mM MES (pH 5.88), 1 mM MgCl_2_. Insoluble CW was pelleted and the supernatant pH was adjusted. CW preparation and CW fragments were treated with RNAse A (Fermentas, http://www.fermentas.com). L. monocytogenes CW fragments, 100 μg/ml MDP (Calbiochem, http://www.emdbiosciences.com/html/CBC/home.html) and 100 μg/ml poly(I:C) (Invivogen), were delivered to macrophage cytosol with Lipofectamine 2000 (Invitrogen).

### Q-RT-PCR.

RNA was harvested from macrophages at 6 h.p.i. using RNeasy Mini kit (Qiagen, http://www.qiagen.com). To synthesize cDNA, 1 μg of total RNA, M-MLV reverse transcriptase, Random Primers, and RNaseOUT ribonuclease inhibitor (Invitrogen) were used. For regular PCR analysis, 1 μg of cDNA was used with specific primers. SYBR Green−based quantitative PCR amplification was performed in 96-well plates using SYBR Green PCR core reagents (Applied Biosystems, http://www.appliedbiosystems.com), the Stratagene Mx3000P Real-Time PCR System (http://www.stratagene.com, and a 60 °C annealing temperature. For each indicated gene, as well as to the reference gene (actin), a standard curve was generated to calculate the quantity of mRNA as function of the Ct value. The level of expression of each gene was determined by normalizing its mRNA quantity to the quantity of the actin mRNA at the same sample. The following mouse primer sequences were designed using Applied Biosystems Primer Express software: *ifnβ*-F: 5′-ctggagcagctgaatggaaag; *ifnβ*-R: 5′-cttgaagtccgccctgtaggt; β-actin-F: 5′-aggtgtgatggtgggaatgg; β-actin-R: 5′-gcctcgtcacccacatagga; *tnfα*-F: 5′-gcaccaccatcaaggactcaa; and *tnfα*-R: 5′-tcgaggctccagtgaattcg; *il-6*-F: 5′-ttccatccagttgccttcttg; and *il-6*-R: 5′-gaaggccgtggttgtcacc; *il-12p40*-F: 5′- aaccatctcctggtttgcca; and *il-12p40*-R: 5′- cgggagtccagtccacctc.

### Measurement of IFN-β.

IFN-β was measured by a mouse IFN-β enzyme-linked immunoassay (ELISA) kit (R&D Systems, http://www.rndsystems.com).

### Labeling of bacteria and immunofluorescence.

Bacterial PGN was labeled as described by Daniel and Errington [41]. A mixture of 1:1 FL-Van (Molecular Probes, http://probes.invitrogen.com) and unlabeled vancomycin (Sigma) was added to growing cultures to a final concentration of 1 μg/ml. The culture was then incubated for 30 min at 37 °C to allow absorption of the antibiotic and then washed four times in PBS. For immunofluorescence microscopy, FL-Van–labeled bacteria or GFP-expressing bacteria were used to infect macrophages on 18-mm^2^ glass cover slips. LysoTracker RED staining was performed according to manufacturer instructions (Molecular Probes). Then, 4 h.p.i., macrophages were washed once with PBS and fixed in 4% paraformaldehyde. Cover slips were incubated with coumarin-phalloidin or tetramethylrhodamine B isothiocyanate-phalloidin (Sigma, 1:1,000 dilution) for cytosolic F-actin staining and mounted with Vectashield mounting medium with DAPI (Vector Laboratories, http://www.vectorlabs.com). Samples were viewed at ×600 with a Nikon TE300 inverted microscope.

## Supporting Information

Figure S1 IFN-β Induction in Peritoneal Macrophages and Intracellular Growth Curve of LLO- Minus Mutant in MyD88-Deficient Macrophages (A) Real-time PCR analysis of IFN-β induction by activated and non-activated resident peritoneal macrophages infected with w.t. L. monocytogenes and LLO-minus mutant.
(B) Intracellular growth curve of LLO-minus mutant in MyD88-deficient BMD macrophages with and without IFN-γ treatment.
(148 KB PDF)

Table S1 The Most Highly Induced Genes by BMD Activated Macrophages Infected with the LLO-Minus Mutant MEEBO array analysis of the complete transcription response of activated and non-activated macrophages to infection with LLO-minus mutant, at 5 h.p.i. Gene induction in activated macrophages was normalized to non-activated macrophages, and the 20 most highly induced genes in activated macrophages infected with LLO-minus mutant are presented.
(13 KB PDF)

### Accession Numbers

MEEBO (http://meebo.ucsf.edu:8080/meebo/meeboQuery.html) gene ID numbers for the genes and gene products discussed in this paper are 5′ nucleotidase (mMC016279); C-C receptor like 2 (mMC024156); C-X-C ligand 1 (mMC023634); CXC ligand 11 (mMC011370); dual specificity phosphatase 16 (mMA034830); IFN activated gene 205 (mMA032762); IFN-β (mMC16397); IL-1α (mMR001431); IL-1 receptor agonist (mMC015067); IL-4 (mMC019427); IL-12 (mMC018187); IL-15 (mMC009424); IL-15 receptor (mMA033400); Mda5 helicase (mMC010553); myxovirus resistance gene (mMC023295); NF-κB light polypeptide zeta (mMC002655); NOD2 receptor (mMR030202); TNFα-induced protein 2 (mMC011682); and TNF receptor super-family member 5 (mMR028074).

## Abbreviations

BMD: bone marrow–derived
CARD: caspase recruitment domain
CW: cell wall
FL-Van: fluorescent vancomycin
GFP: green fluorescent protein
h.p.i.: hour post-infection
IFN: interferon
IL: interleukin
IRF: interferon regulatory factor
LLO: listeriolysin O
MDP: muramyl dipeptide
MEEBO: Mouse Exonic Evidence Based Oligonucleotide
NOD: nucleotide-binding oligomerization domain
PGN: peptidoglycan
Q-RT-PCR: quantative real-time PCR
TLR: Toll-like receptor
TNF: tumor necrosis factor
V-ATPase: vacuolar ATPase
w.t.: wild-type
