# Supplementary figures and images for: Bacterial Ligands Generated in a Phagosome Are Targets of the Cytosolic Innate Immune System
Source: PLoS Pathog. 2007 Mar 30;3(3):e51. doi: 10.1371/journal.ppat.0030051 (PMC1839167; doi:10.1371/journal.ppat.0030051)

A.

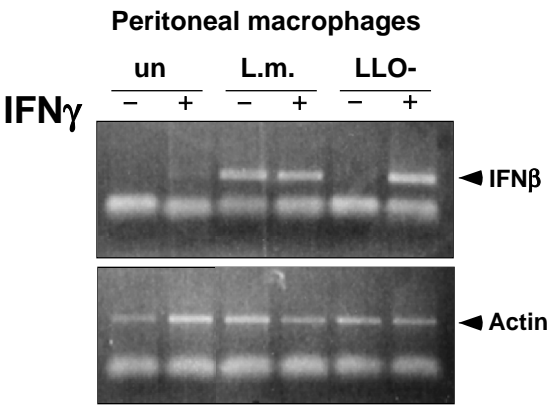

B.

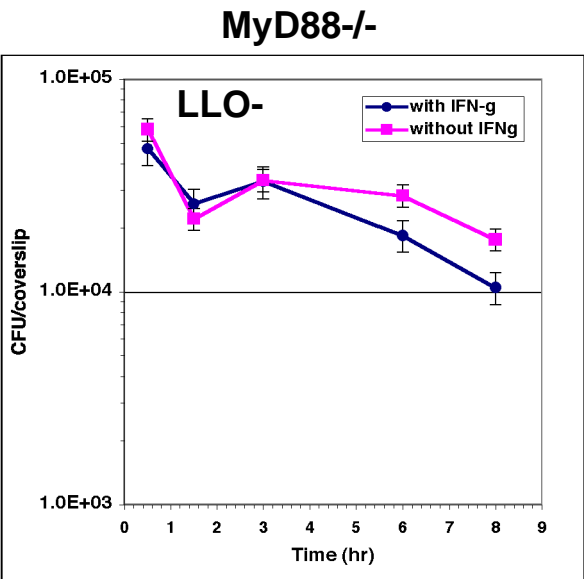

Supplement: Figure S1 — (A) Real-time PCR analysis of IFN-β induction by activated and non-activated resident peritoneal macrophages infected with w.t. L. monocytogenes and LLO-minus mutant. (B) Intracellular growth curve of LLO-minus mutant in MyD88-deficient BMD macrophages with and without IFN-γ treatment. (148 KB PDF) [file ppat.0030051.sg001.pdf]
