# Supplementary material for: Bacterial Ligands Generated in a Phagosome Are Targets of the Cytosolic Innate Immune System
Source: PLoS Pathog. 2007 Mar 30;3(3):e51. doi: 10.1371/journal.ppat.0030051 (PMC1839167; doi:10.1371/journal.ppat.0030051)
Supplement: Table S1 — MEEBO array analysis of the complete transcription response of activated and non-activated macrophages to infection with LLO-minus mutant, at 5 h.p.i. Gene induction in activated macrophages was normalized to non-activated macrophages, and the 20 most highly induced genes in activated macrophages infected with LLO-minus mutant are presented. (13 KB PDF) [file ppat.0030051.st001.pdf]

Herskovits et al., Supplementary data, table S1

Table S1: Most highly Induced genes in activated macrophages infected with LLO-minus mutant:

MEEBO ID	Gene name	Log2 induction
mMC015165	nitric oxide synthase 2	6.593
mMC013933	c-mer proto-oncogene tyrosine kinase	5.396
mMC003369	kinase insert domain protein receptor	4.999
mMR029868	Casitas B-lineage lymphoma b	4.952
mMC024100	pleckstrin homology, Sec7 and coiled-coil	4.894
mMR030627	c-mer proto-oncogene tyrosine kinase	4.811
mMC007730	protein C receptor	4.591
mMC018187	interleukin 12b	4.486
mMC006264	Casitas B-lineage lymphoma b	4.31
mMC024156	chemokine (C-C motif) receptor-like 2	4.206
mMC016514	myosin regulatory light chain	4.205
mMC017125	Casitas B-lineage lymphoma b	4.172
mMC016397	interferon beta 1	3.937
mMC009298	interleukin 27	3.881
mMC007760	ubiquitin D	3.873
mMC024492	sterol O-acyltransferase 1	3.631
mMC013863	nuclear factor of kappa light polypeptide	3.591
mMC022207	stannin	3.473
mMA032885	interleukin 1 receptor	3.441
mMC015067	interleukin 1 receptor antagonist	3.405

*MeeboDB gene annotation database:
http://meebo.ucsf.edu:8080/meebo/meeboQuery.html
